# Antigen-dependent interplay of formulation, systemic innate responses, and antibody responses to multi-component replicon RNA vaccination

**DOI:** 10.1016/j.omtn.2025.102595

**Published:** 2025-06-09

**Authors:** Taishi Kimura, Steven J. Reed, Nikole L. Warner, Megan N. Fredericks, Thomas B. Lewis, Allie Lafferty, Edgar Hodge, Adrian Simpson, Troy Hinkley, Amit P. Khandhar, Deborah H. Fuller, Jesse H. Erasmus

**Affiliations:** 1 HDT Bio, Seattle, WA 98109, USA; 2 Department of Microbiology, University of Washington, Seattle, WA 98109, USA; 3 Washington National Primate Research Center, University of Washington, Seattle, WA 98121, USA

**Keywords:** MT: Delivery Strategies, replicon RNA, lipid nanoparticles, RNA vaccines, innate immunity, type I interferon, enterovirus, respiratory syncytial virus, cationic nanocarrier, lipid nanoparticle

## Abstract

Replicon RNA (repRNA) vaccines are a transformative tool in combating infectious diseases, eliciting robust immune responses at lower doses. However, systemic inflammatory responses to lipid nanoparticle (LNP)-formulated RNA vaccines may compromise safety and efficacy. repRNA vaccines delivered with cationic nanocarriers like LION offer a promising alternative by localizing innate immune responses required to induce a robust adaptive response at the site of injection. Here, we show, in pigtail macaques, that a multi-component repRNA vaccine against two viral pathogens formulated with LION induces antigen-specific antibody (Ab) responses against enterovirus D68 (EV-D68) while reducing systemic inflammatory responses compared to the same vaccine formulated with LNP. Notably, early systemic interferon (IFN) levels inversely correlate with EV-D68 binding and neutralizing Ab titers, indicating that excessive systemic innate immune responses can impair RNA vaccine immunogenicity. These findings suggest that LION-mediated delivery offers a safer and more effective platform for RNA vaccines than conventional LNP formulations. By mitigating systemic cytokine induction, LION enhances vaccine immunogenicity and safety—key considerations for optimizing RNA vaccine design.

## Graphical abstract

**Figure:**
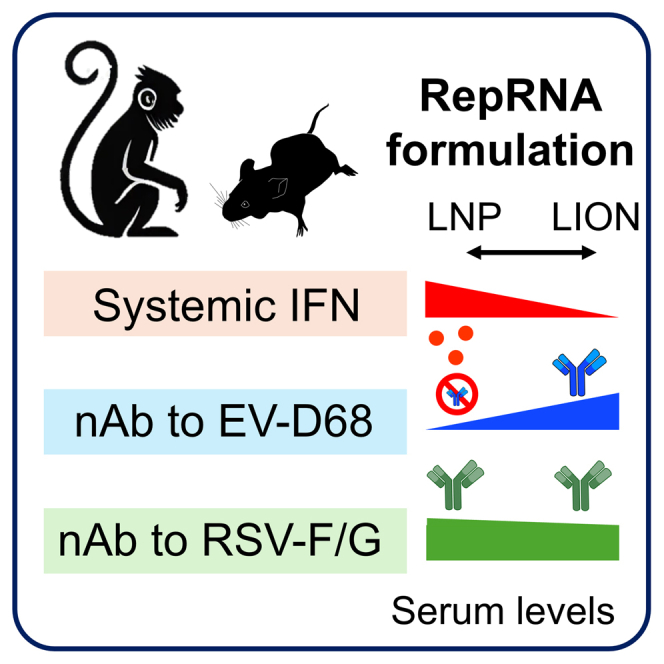

## Teaser

Kimura, Erasmus, and colleagues demonstrate that systemic innate responses inversely correlate with antibody responses against enterovirus D68—but not RSV—following multi-component replicon RNA immunization. Their findings show that formulation-dependent innate activation shapes antigen-specific outcomes, positioning LION as a promising strategy to improve safety and immunogenicity of RNA vaccines.

## Introduction

Replicon RNA (repRNA) vaccines have significant potential to counteract infectious diseases by eliciting robust adaptive immune responses at lower doses than conventional mRNA vaccines.^1^^,^^2^^,^^3^^,^^4^^,^^5^^,^^6^^,^^7^ Nevertheless, early clinical trials using repRNA formulated with lipid nanoparticle (LNP) (repRNA/LNP) showed that even at a dose of 10 μg—3 to 10 times lower than the typical doses of conventional RNA vaccines—80%–100% of participants experienced systemic reactogenicity, including grade 3 adverse events.^4^^,^^5^ In contrast, a phase II clinical trial using repRNA formulated with a cationic nanocarrier reported that only 12.9% of participants experienced any adverse events at 10 μg dose, with no unsolicited or serious adverse events reported by the end of the study.^3^ Therefore, the observed difference in clinical trials using different delivery methods suggests that formulation plays a key role in determining reactogenicity. We recently conducted a comparative study in mice using repRNA/LNP and repRNA formulated with a cationic nanocarrier, LION (repRNA/LION).^8^ These two formulations exhibited distinct biodistribution: as previously reported by others, repRNA/LNP was distributed broadly across multiple organs, including the liver, spleen, and lungs,^9^ whereas repRNA/LION remained localized in the muscle and draining lymph nodes (dLNs). The subsequent innate immune responses mirrored this differential biodistribution: repRNA/LNP triggered systemic innate responses, including excessive type I IFN responses in the liver, muscle, and dLNs, along with elevated serum cytokine production as previously reported by others,^10^^,^^11^ whereas repRNA/LION induced a robust local innate response, as evidenced by cytokine and IFN-related gene expression in the muscle and dLNs and significantly reduced systemic inflammatory responses compared to repRNA/LNP. In addition, mice injected with repRNA/LNP exhibited greater weight loss and a transient elevation in serum cardiac troponin I levels—a well-established serological marker of cardiac damage—within 4 h of intramuscular immunization, whereas mice injected with repRNA/LION showed only minimal changes. Given that systemic innate responses are closely associated with systemic reactogenicity,^12^^,^^13^ the absence of aberrant systemic cytokine responses and weight loss in repRNA/LION-immunized mice may explain the clinically observed high tolerability when repRNA is formulated with cationic nanocarriers. Furthermore, *in vivo* delivery of a multi-component repRNA, composed of an antigen from enterovirus D68 (EV-D68) and two additional antigens from other viruses to increase the total dose, by LION induced a protective response against EV-D68 through induction of a neutralizing Ab response.^8^^,^^14^ In contrast, the same combination of multi-component repRNA/LNP failed to induce an EV-D68-neutralizing Ab response despite inducing robust systemic cytokine production and binding Abs to the other vaccine components. To validate these findings in primates, we performed a follow-up study in pigtail macaques.

## Results

### RepRNA/LION does not induce excess systemic cytokine responses in pigtail macaques

**Figure 1:**
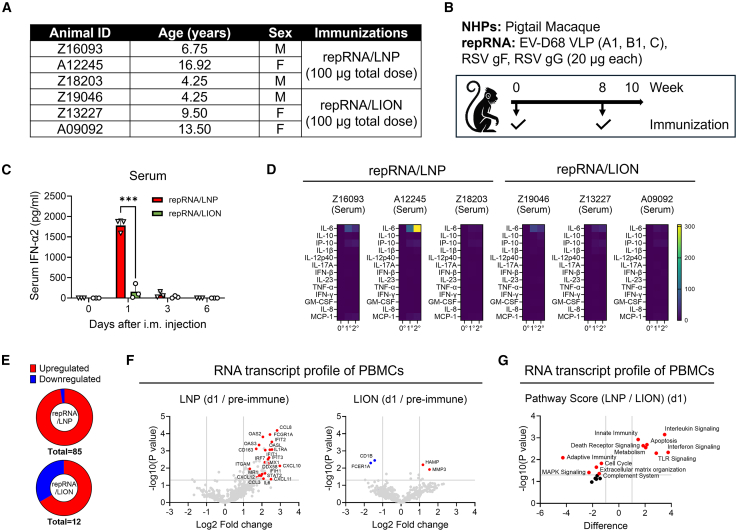
RepRNA/LION induces minimal systemic cytokines in NHPs (A) Experimental groups: six pigtail macaques were divided into two groups and immunized with either repRNA/LNP or repRNA/LION formulations (*n* = 3 per group). (B) Experimental plan: macaques were intramuscularly injected with multi-component repRNA encoding three virus-like particles (VLPs) of enterovirus D68 (EV-D68) (subclades A1, B1, and C) and two glycoproteins (gF and gG) of respiratory syncytial virus (RSV). Each repRNA (20 μg) was formulated with LNP or LION and mixed to yield a total dose of 100 μg. Immunizations were administered at weeks 0 and 8, with blood samples collected at weeks 0, 1, 3, 6, 8, and 10. (C) Serum IFN-α levels at indicated time points post-immunization, determined by ELISA. (D) Cytokine bead assay results for indicated cytokine levels in sera at baseline (week 0), 16 h post-prime (1°), and 16 h post-boost (2°). Results for repRNA/LNP (left) and repRNA/LION (right) groups are shown as a heatmap. (E–G) Nanostring analysis of PBMCs isolated at day 1 post-prime immunization compared to those isolated before immunization (pre-immune). (E) Circle chart showing the differential regulation of transcripts (*p* < 0.05) in response to repRNA/LNP or repRNA/LION. (F) Volcano plots with representative upregulated (red) and downregulated (blue) genes. RNA transcripts from PBMCs isolated before immunization (pre-immune) were used as the baseline for the calculation. (G) Pathway scores calculated from RNA transcripts of PBMCs isolated from macaques receiving repRNA/LNP, relative to those receiving repRNA/LION. Scores are presented as the difference in Z-transformed values between the two groups. Positive values on the x-axis indicate pathways with higher expression in PBMCs from the LNP group compared to the LION group. Error bars indicate mean ± SD. Each dot represents the value of an individual. Statistical significance was determined by unpaired (C) and paired (F) Student’s two-tailed t test. Statistically significant results are denoted as *p* < 0.05∗, <0.01∗∗, <0.001∗∗∗, and <0.0001∗∗∗∗.

To determine whether our observation that repRNA/LION induces minimal systemic cytokine production in mice is translatable to non-human primates (NHPs), we enrolled six pigtail macaques, divided into two groups; one received repRNA/LNP and the other received the same repRNA formulated with LION (Figure 1A). Twenty micrograms each of repRNA-encoding virus-like particles (VLPs) of three distinct EV-D68 genotypes (subclades A1, B1, and C)^14^ and two additional antigens from respiratory syncytial virus (RSV) (RSV-F and RSV-G) were formulated with LNP or LION and mixed to yield a 100-μg total dose of multi-component repRNA. Including EV-D68 irrelevant antigens (RSV-F and RSV-G) allows us to (1) amplify innate immune responses by increasing the total RNA dose beyond that of each individual monovalent repRNA component and (2) assess antigen-specific effects within a shared, robust innate immune context, across three distinct antigen classes, including non-enveloped VLPs (EV-D68), as well as type I (RSV-F) and type II (RSV-G) glycoproteins of enveloped viruses. Since IFN responses following intramuscular injection of repRNA/LNP are known to be dose-dependent,^15^ we selected a 100 μg dose to ensure strong systemic cytokine induction. Macaques were intramuscularly immunized twice, 8 weeks apart (Figure 1B). As previously observed in mice,^8^^,^^11^ serum IFN-α2 levels rose within ∼16 h post-immunization in repRNA/LNP-injected macaques but increased only modestly in repRNA/LION-injected macaques (approximately 11.3-fold difference in the mean values between the two groups) (Figure 1C). A cytokine bead assay showed most serum cytokine levels, including another subtype of type I IFN (IFN-β) and type II IFN (IFN-γ), remained stable (Figure 1D). However, one macaque receiving repRNA/LNP exhibited a 305-fold increase in interleukin-6 (IL-6) following the second dose, whereas those receiving repRNA/LION showed an utmost increase of 42.4-fold in IL-6 levels (Figure 1D). Therefore, IFN-α2 is the primary cytokine induced systemically by repRNA/LNP in macaques at day 1. Type I IFN signaling (e.g., IFN-α/IFN-β) triggers the expression of antiviral genes.^16^^,^^17^ Therefore, we next extracted RNA from isolated peripheral blood mononuclear cells (PBMCs) one day after immunization and performed NanoString analysis. RepRNA/LNP upregulated 83 genes in PBMCs; in comparison, repRNA/LION upregulated approximately 10-fold fewer genes (eight genes) (Figure 1E). Highly upregulated genes include those of chemokines and IFN-inducible genes, and the levels were much higher in macaques receiving repRNA/LNP than those receiving repRNA/LION (Figure 1F). Pathway scoring analysis revealed that many pathways were more prominently expressed in PBMCs from NHPs receiving repRNA/LNP compared to those receiving repRNA/LION (Figures 1G and S1). These pathways included IFN signaling, IL signaling, Toll-like receptor (TLR) signaling, and innate immunity. In contrast, genes associated with adaptive immunity were more highly expressed in PBMCs from NHPs that received repRNA/LION than in those that received repRNA/LNP (Figures 1G and S1). Thus, NHPs receiving repRNA/LION exhibit significantly lower systemic cytokine responses compared to those receiving repRNA/LNP, particularly in IL and IFN signaling, while maintaining high levels of genes associated with adaptive immunity in PBMCs, likely primed locally in the injected muscle and dLNs.

### RepRNA/LION induces antigen-dependent Ab responses in NHPs following multi-component immunization

**Figure 2:**
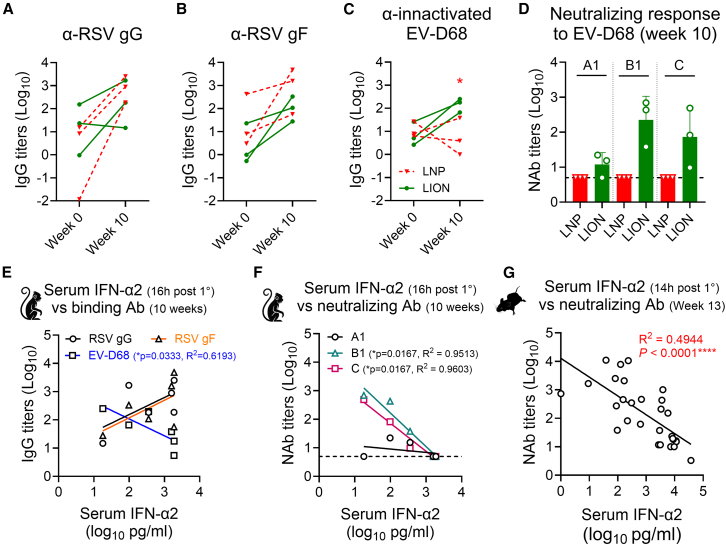
Systemic cytokine levels inversely correlated with anti-EV-D68 Ab titers (A–C) Total IgG titers (Log_10_ EC50 values) in macaques immunized with repRNA/LNP (red) or repRNA/LION (green) at week 0 (dashed lines) and week 10 (solid lines), as determined by ELISA. Upper panels: mean optical density (OD) values at 450 nm. Antibody responses to specific antigens are shown: (A) RSV-G, (B) RSV-F, and (C) inactivated EV-D68. (D) Neutralizing antibody (NAb) responses against EV-D68 subclades (A1, B1, and C) in sera from macaques immunized with repRNA/LNP (red) or repRNA/LION (green). Log10 values of the 50% reciprocal NAb titers for the indicated subclades of EV-D68 are shown. (E–G) Correlation analysis results between immune parameters and antibody responses in macaques and C57BL/6 mice: (E) serum IFN-α levels at day 1 post-prime dose versus total IgG levels to RSV-G (black), RSV-F (orange) and inactivated EV-D68 (blue) at week 10 in macaques receiving repRNA/LNP or repRNA/LION. (F) Serum IFN-α levels at day 1 post-prime dose versus neutralizing titers to EV-D68 subclades (A1, B1, and C) in macaques receiving repRNA/LNP or repRNA/LION. (G) Serum IFN-α2 levels at 14 h post-prime dose versus neutralizing titers to EV-D68 subclade B2 in C57BL/6 mice. Pooled data from mice receiving monovalent, multi-component repRNA formulated with both LION and LNP are included. Each dot represents the value of an individual. Lines indicate simple linear regression results. Statistical significance was determined using Spearman correlation analysis, with significant results denoted as *p* < 0.05∗, <0.01∗∗, <0.001∗∗∗, and <0.0001∗∗∗∗.

We next measured the antigen-dependent Ab response to recombinant RSV-G and RSV-F proteins, as well as inactivated EV-D68 in sera of immunized macaques by ELISA. At week 10, 2 weeks after the booster dose, binding Ab responses to RSV-G (Figure 2A) and RSV-F (Figure 2B) were almost equally induced in macaques receiving repRNA/LION and those receiving repRNA/LNP, reproducing our previously published result in mice.^8^ Notably, the Ab response to EV-D68 was only induced in macaques receiving repRNA/LION, but not in those receiving repRNA/LNP (Figure 2C). Consequently, no neutralizing Ab titers were detected in macaques receiving repRNA/LNP against three examined subclades (A1, B1, and C) of EV-D68 (Figure 2D). The absence of the neutralizing Ab response in multi-component repRNA/LNP-injected macaques is consistent with our previous results in mice.^8^ Therefore, while multi-component repRNA/LNP failed to induce an Ab response to EV-D68, LION enabled an Ab response to all antigen components encoded in the multi-component repRNA mixture.

### Systemic innate responses negatively correlate with anti-EV-D68 neutralizing Ab titers

The formulation-dependent difference in the systemic cytokine levels and α-EV-D68 Ab levels in mice^8^ and macaques (Figures 1 and 2A–2D) led us to analyze the overall correlation between these two parameters independent of vaccine formulations. To do this, we first plotted the serum IFN-α2 levels against binding Ab titers to RSV-G, RSV-F, and inactivated EV-D68. Although no significant correlations were observed between α-RSV Abs and serum IFN-α2 levels, the α–EV-D68 Ab response showed a significant negative correlation (Figure 2E). Next, we plotted the serum IFN-α2 levels against neutralizing Ab titers against three subclades of EV-D68. Although neutralizing Ab titers to EV-D68 (subclade A1) did not correlate with serum IFN-α2 levels, those to B1 and C subclades showed a significant negative correlation (Figure 2F). To confirm whether this finding applies to the mouse model, we replotted our historic mouse neutralizing Ab data and serum IFN-α2 levels, including both monovalent and multi-component immunized groups.^8^ Spearman correlation analysis showed a strong negative correlation between these two parameters (Figure 2G). These data suggest that early (<1 day) serological testing for IFN responses after repRNA immunization may be useful for predicting neutralizing Ab responses to EV-D68 in the context of non-enveloped VLP-based repRNA-encoded antigens. Overall, our data demonstrate that multi-component repRNA/LION vaccines could be an effective tool to mitigate the potential interference that a systemic cytokine response can have on RNA-encoded antigens, thereby enabling Ab responses to each vaccine component in high-dose multi-component vaccine mixtures.

## Discussion

The current study corroborates our previous findings in mice, demonstrating that repRNA vaccines delivered via LION elicit minimal systemic cytokine responses due to their localized biodistribution, whereas those delivered via LNP induce robust dose-dependent systemic responses by circulating throughout the body.^8^ This observation extends to NHPs. Similarly, we confirmed in NHPs that repRNA/LNP fails to induce an Ab response to EV-D68 when mixed with repRNA-encoding non-EV-D68 antigens (in this study, RSV-G and RSV-F), resulting in a high overall repRNA dose (100 μg in this case), concurrent with early transient systemic IFN-α2 responses. In contrast, our previous studies using monovalent repRNA/LNP successfully induced neutralizing Ab responses to EV-D68.^8^^,^^14^ Because an effective EV-D68 repRNA vaccine will likely require (1) targeting of multiple antigenic variants and (2) induction of neutralizing Ab responses,^18^ controlling systemic cytokine responses to high-dose multi-component repRNAs may be crucial.

Early and transient systemic cytokine production, as observed in NHPs receiving repRNA/LNP, is a well-known feature of viral infections, including those caused by alphaviruses and enteroviruses.^19^^,^^20^ In particular, IFNs play a critical role in viral clearance during the early phase of infection.^21^ However, sustained cytokine responses have been associated with T cell exhaustion and immune-mediated tissue damage, so-called immunopathology.^21^ Therefore, IFN induction must be tightly regulated and transient during viral infection. Our current findings suggest that repRNA/LNP immunization similarly induces systemic transient IFN responses and that these responses may differentially influence the immunogenicity of specific antigens. Future studies using a preconditioned model with IFNs will allow us to further analyze the impact of increased systemic IFN levels on neutralizing Ab responses to repRNA vaccines.

The antigen-specific and subclade-specific negative correlation may reflect the sensitivity of individual RNAs and the encoded antigens to antiviral cytokines, including IFNs. IFNs inhibit various stages of the viral life cycle, including interference with viral mRNA translation, disruption of structural protein trafficking, promotion of viral RNA degradation, and acceleration of viral protein degradation.^22^ These effects collectively reduce the availability of antigens for presentation, potentially impairing the induction of neutralizing Ab responses. Notably, a recent study demonstrated that co-delivery of antigen-encoding repRNA with siRNA targeting IFNAR1 via LNP enhanced germinal center formation and Ab responses.^23^ These findings suggest an inhibitory role of type I IFN in the germinal center response, which could explain the reduced neutralizing Ab responses observed against B1 and C subclades of EV-D68 in the present study. While RSV-F and RSV-G are each expressed as type I and type II glycoproteins, respectively, assembling into multimeric structures at the plasma membrane, EV-D68 VLPs require more complex expression, involving cytoplasmic co-expression of virus structural proteins and nonstructural proteases, the latter mediating processing, assembly, and release of non-enveloped particles via a poorly understood mechanism. This added complexity may make EV-D68 VLP antigens more susceptible to inhibition by IFN or other cytokine-mediated mechanisms compared to RSV-F and RSV-G. The differential sensitivity of each repRNA-encoded VLP subclade of EV-D68 to systemic IFN may reflect (1) viral immune evasion strategies encoded in their RNAs or antigens,^24^^,^^25^ (2) a result from selective innate recognition by immune sensors influenced by sequence-dependent structural elements,^26^ or (3) differential expression and VLP assembly/release efficiency. Consequently, elevated systemic IFN levels in the repRNA/LNP group could disrupt effective antigen assembly and processing, contributing to the impaired neutralizing Ab responses observed against EV-D68 subclades B1 and C. PBMCs from macaques immunized with repRNA/LNP, compared to those receiving repRNA/LION, showed higher expression of several innate immune-response-related pathways, including IL signaling, TLR signaling, general innate immunity, and IFN signaling. This suggests that additional cytokines may have been transiently induced prior to day 1 post-immunization with repRNA/LNP. Regardless of whichever cytokines other than IFN are involved, multi-component repRNA/LION still induced neutralizing Ab responses in NHPs without triggering robust systemic innate responses, highlighting the resistance of this platform for such an inhibitory pathway.

Several studies have emphasized the importance of systemic innate responses as prerequisites for adaptive immune activation when utilizing repRNA with LNP formulations.^7^^,^^10^^,^^11^^,^^15^ However, our findings across species (rodents and primates) suggest that localized innate responses are more critical for immunogenicity than systemic innate responses.^8^ Localized repRNA/LION delivery appears to mitigate systemic cytokine responses, thereby preserving neutralizing Ab response pathways that might otherwise be suppressed. Similar observations have been also reported by others using muscle-targeting LNPs, as well as jet injection of naked mRNA, both showing localized biodistribution and minimal systemic cytokine induction, yet still inducing robust immune responses.^27^^,^^28^

Systemic cytokine responses are closely associated with systemic reactogenicity,^12^^,^^13^ and cytokine genes preferentially upregulated in the PBMCs of NHPs receiving repRNA/LNP, such as CXCL10 and IL1RA, have also been reported to be upregulated in a cohort of patients who developed myocarditis/pericarditis following mRNA/LNP vaccination.^29^ While systemic cytokine levels do not always reflect tissue inflammation, monitoring these responses could guide the refinement of repRNA formulations to optimize EV-D68 vaccines for both immunogenicity and safety, which are not yet available in clinical practice.

## Materials and methods

### Animal models and ethical considerations

All animal studies were approved by the University of Washington’s Institutional Animal Care and Use Committee (IACUC). The Washington National Primate Research Center where the animal studies were conducted is accredited by the American Association for the Accreditation of Laboratory Animal Care (AAALAC).

Non-human primates (NHPs): age, sex, and source of the pigtail macaques used in the study are included in Figure 1A.

### Cells and viruses

Rhabdomyosarcoma (RD) cells were purchased from the American Type Culture Collection (ATCC, Cat# CCL-136) and grown in complete media containing DMEM with high glucose (Gibco, Cat# 11995) supplemented with 10% fetal bovine sera (FBS) and 1% Pen/Strep (Gibco, Cat# 15140). Cells were maintained at 37°C and 5% CO2. One representative isolate from each clade of EV-D68 was obtained from the Biodefense and Emerging Infections Research Resources Repository (BEI Resources). The isolates include the following: subclade A1: USA/WI/2009–23230 (BEI, NR-51995), subclade B1: US/MO/14–18947 (NR-49129), and subclade C: USA/2003–23226 (NR-51990). Virus stocks were grown in RD cells at 33°C with 5% CO2. Confluent flasks of RD cells were infected at a MOI of 0.1 with EV-D68 stocks from BEI and incubated until the cytopathic effect was observed, about 2 days post-infection. These master stocks were then titrated by plaque assay and used to propagate working stocks used for subsequent experiments.

### Vaccines and formulations

VLPs for EV-D68 subclade A1 (GenBank: AGC00381), subclade B1 (GenBank: AKU75617), subclade C (GenBank: BAK08579), RSV-F (GenBank: AIO08046), and RSV-G (RefSeq: YP_009518856) proteins were subcloned and used as repRNA-encoded antigens. repRNA was enzymatically synthesized using T7 polymerase, followed by DNase I treatment and capping with a Vaccinia enzyme to yield Cap-0 RNA. All RNAs were precipitated with lithium chloride, and purified with ethanol, and quality-checked for integrity by capillary gel electrophoresis (Agilent 5200 Fragment Analyzer System), purity, and concentration (Nanodrop 2000) as described previously.^8^^,^^14^ LION formulation was prepared as previously described using emulsification and microfluidization, achieving a particle size of ∼60 nm (PDI ≤0.2). LION was sterile-filtered and stored at 2°C–8°C. LION was formulated with each monovalent repRNA first at N:P ratio of 15, then mixed before immunization. LNPs were formulated individually as monovalent vaccines and combined prior to injection. Monovalent LNPs were prepared by mixing RNA in citrate buffer (pH 4.5) with lipids (SM-102:DSPC:Cholesterol:DMG-PEG 2000 at 50:10:38.5:1.5) in ethanol using a micromixer chip (Dolomite Microfluidics), followed by overnight dialysis against PBS. repRNA/LION and repRNA/LNP formulations were prepared for each immunization and used within 4 and 72 h, respectively. Although the stability of repRNA/LNP used in this specific study was not directly tested, prior internal assessments have demonstrated no detectable degradation in RNA integrity, no changes in particle size or PDI, and stable RNA incorporation at 4°C over a 120-h period under similar formulation and storage conditions. Monovalent LNPs were seen to have >95% encapsulation efficiency assessed via RiboGreen assay, particle sizes between 85 and 115 nm, PDIs between 0.12 and 0.25, and zeta potentials between 13 and 38 mV as determined by Malvern Zetasizer. Both LION and LNPs were stored in optimal conditions as previously described.^8^

### Immunization

For dosing, 100 μg of multi-component repRNA formulated with LION or LNP was injected intramuscularly into pigtail macaques twice 8 weeks apart from each immunization. Detailed group division is included in Figure 1B.

### Whole blood processing

For serum separation, blood was collected in BD Vacutainer SST tubes and centrifuged to isolate serum. For PBMC isolation, blood was collected in BD Vacutainer EDTA tubes and centrifuged to isolate plasma. PBMCs were further isolated from remaining blood using Histopaque-1077 (Millipore Sigma, Burlington, MA, Cat#10771). After centrifugation, PBMCs were carefully removed. Red blood cells, if visible, were removed using ACK lysing buffer. PBMCs were counted using a Nexcelom cellometer (Nexcelom Bioscience, Lawrence, MA). Cells were resuspended in freezing media (FBS+10% DMSO) and then placed in a Mr. Frosty container at −80°C overnight and then transferred to liquid nitrogen freezer for long-term storage.

### Cytokine and immune response analysis

RNA was isolated from these PBMCs using RNeasy Mini Kit (QIAGEN, Cat# 74104) according to the manufacturer’s instructions. One hundred nanograms of purified RNA was assessed for gene expression using the nCounter NHP Immunology Panel cartridge (NanoString Technologies, Cat# 115000276) on the nCounter Sprint instrument. Data were analyzed with NanoString nSolver software using standard analysis (Quality Control and Data normalization) and advanced analysis (Pathway Scoring) modes and visualized by GraphPad Prism. For serum cytokine analysis, sera were separated from Microvette 200 CAT-Gel tubes (Sarsteder, Numbrecht, Germany Cat# 20.1291) by centrifuging at 10,000 rpm for 5 min at room temperature. Serum cytokine levels were analyzed by Cynomolgus/Rhesus IFN-Alpha ELISA Kit (PBL Assay Sciences, Cat# 46100) or LEGENDplex NHP Inflammation Panel (BioLegend, San Diego, CA, Cat# 740389) according to the manufacturer’s instructions. Flow cytometry data were acquired on a FACSymphony A3 flow cytometer (BD). The acquired data were analyzed using FlowJo software (BD). LEGENDplex data were analyzed using LEGENDplex Data Analysis Software Suite (Qognit).

### Ab response analysis

Inactivated EV-D68 was used for EV-D68 binding. Immunoglobulin G (IgG) ELISA EV-D68 isolate USA/WI/2009–23230 was purified and inactivated as described in Krug et al. (cite Peter Krug et al., 2023, Science Advances). To measure anti-EV-D68 Ab binding titers, a 384-well Corning high bind plate (cat# 3700) was coated with inactivated virus at a concentration of 2 μg/mL in 1xPBS and incubated overnight at 2°C–8°C. Subsequently, the plate was washed (1xPBS, 0.05% Tween) and blocked for 2 h in blocking buffer (1xPBS, 0.05% Tween, 1% nonfat dry milk) while shaking at room temperature (RT) and washed. NHP samples were first diluted 1:10 in blocking buffer, then serially diluted 1:4 in blocking buffer, and subsequently added to the blocked ELISA plate, incubated for 1 h while shaking at RT, and washed. Polyclonal goat anti-monkey IgG HRP (Thermo Fisher, Cat# PA1-84631) was diluted 1:10,000 in blocking buffer and next added to the ELISA plate and incubated, shaking for 1 h at RT and washed. TMB substrate (Seracare, Cat# 5120-0083) was then added and allowed to develop while pre-reading the plate at a wavelength of 630 nm (ELX808, Bio-Tek Instruments Inc). After sufficient substrate development (∼20 min), KPL stop solution was added to quench substrate development, and the plate was read on a spectrophotometer at 450/630 nm. Antigen-specific IgG responses were evaluated by ELISA as previously described.^30^

For binding IgG response analysis, RSV antigens were obtained from the Biodefense and Emerging Infections Research Resources Repository (BEI Resources) and SinoBiological. Briefly, ELISA plates were coated with 2 μg/mL of human RSV A2 F and G proteins (Sino Biological, Cat# 11049-V08B and 40830-V08H, respectively; resources, NR-58648 and NR-59001). Animal sera was serially diluted sand detected via anti-monkey IgG-HRP (Thermo Fisher Cat# PA1-84631). Plates were developed using a TMB substrate (Seracare, Cat# 5120-0083), and absorbance was measured at 450 nm (ELX808, Bio-Tek Instruments Inc). Monkey total IgG concentrations were determined from a standard curve using purified mouse IgG. Neutralizing antibody response was measured as previously described.^8^^,^^14^

### Correlation and statistical analyses

Indicated statistical comparisons, as described in the figure legends, were performed using GraphPad Prism (GraphPad). For correlation studies, log10 transformed data were analyzed by Spearman correlation coefficients, two-tailed with 95% confidence interval. *p* values less than 0.05 were considered significant and are indicated in figures as follows: *∗p* < 0.05; ∗∗*p* < 0.01; ∗∗∗*p* < 0.001; *∗∗∗∗p* < 0.0001.

## Data availability

All data associated with this study are present in the paper or the Supplemental Information. RepRNAs and the LION formulation can be made available under a material transfer agreement upon request to J.H.E.

## Acknowledgments

We thank the WaNPRC and RML animal staff for the excellent care of the animals. This work was supported by NIH grant no. 1R43AI165100 (J.H.E.), P51OD010425 (WaNPRC), and HDT Bio internal funds. A part of this work was supported by NIH/NIAID grant R01AI180195 and by a 2025 Career Development Award from the American Society of Gene & Cell Therapy (T.K.). The content is solely the responsibility of the authors and does not necessarily represent the official views of the funders.

## Author contributions

T.K., S.J.R., N.L.W., E.H., D.H.F., and J.H.E. designed the experiments. T.K., N.L.W., E.H., and S.S. performed the experiments. M.N.F., T.B.L., A.L., T.H., and A.P.K. prepared the samples and materials. T.K., A.P.K., D.H.F., and J.H.E. supervised the experiments and wrote the manuscript.

## Declaration of interests

T.K., S.J.R., N.L.W., A.L., E.H., A.S., T.H., A.P.K., D.H.F., and J.H.E have equity interest in HDT Bio. J.H.E. and A.P.K. are co-inventors on patents (US Patent No. 11,458,209; 11,433,142; 11,752,218; 11,648,321; 11,654,200) and patent applications (PCT/US22/76787, PCT/US23/60225, PCT/US2024/010326) pertaining to the LION formulation and repRNA compositions described in the studies. All other authors declare that they have no competing interests.

## Supplemental information

Document S1. Figure S1

Document S2. Article plus supplemental information
