# Supplementary material for: Antigen-dependent interplay of formulation, systemic innate responses, and antibody responses to multi-component replicon RNA vaccination
Source: Mol Ther Nucleic Acids. 2025 Jun 9;36(3):102595. doi: 10.1016/j.omtn.2025.102595 (PMC12221443; doi:10.1016/j.omtn.2025.102595)
Supplement: Document S1. Figure S1 [file mmc1.pdf]

OMTN, Volume *36*

Supplemental information

Antigen-dependent interplay of formulation, systemic innate responses, and antibody responses to multi-component replicon RNA vaccination

Taishi Kimura, Steven J. Reed, Nikole L. Warner, Megan N. Fredericks, Thomas B. Lewis, Allie Lafferty, Edgar Hodge, Adrian Simpson, Troy Hinkley, Amit P. Khandhar, Deborah H. Fuller, and Jesse H. Erasmus

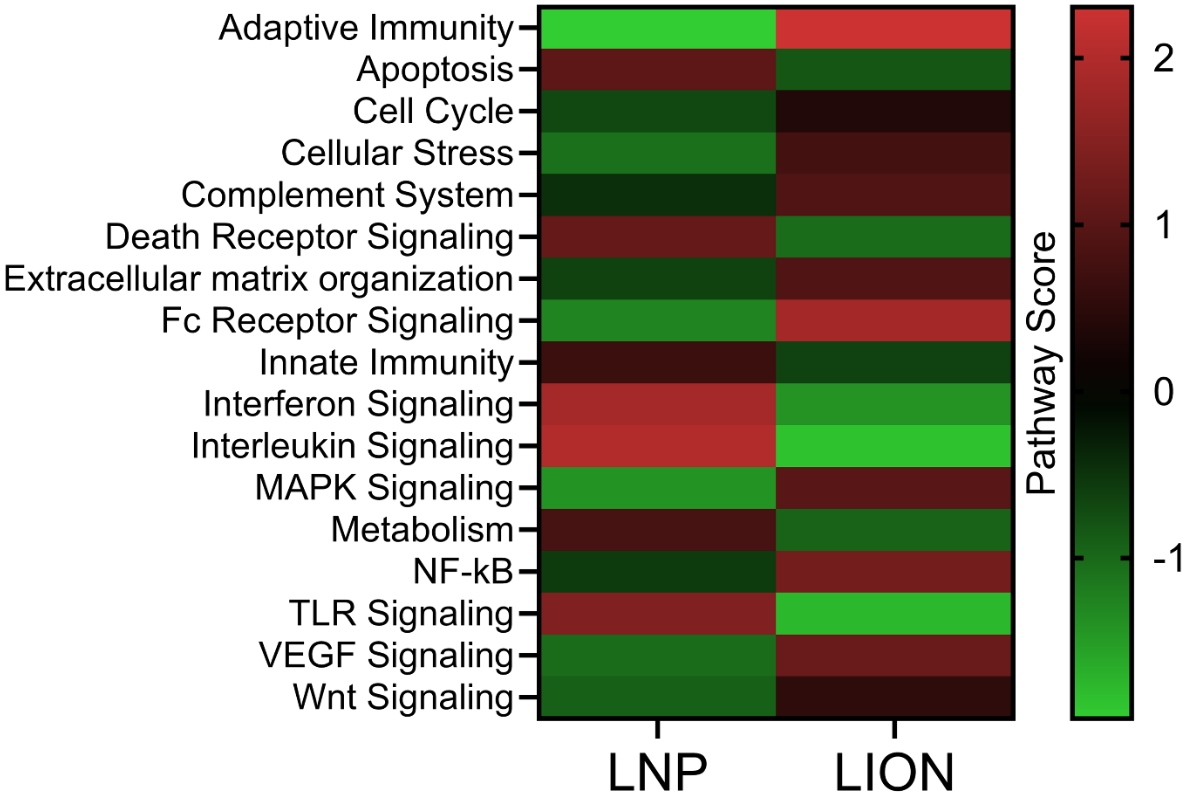

Fig. S1 | Differential pathway expression in PBMCs isolated from NHPs immunized with repRNA/LNP versus repRNA/LION.

Pathway scores of RNA transcripts isolated from PBMCs receiving repRNA/LNP and repRNA/LION. Scores are displayed as the Z-transformed values.
